# Impact of Removing the Monitoring Requirements for Holdings with Atypical Scrapie in Great Britain

**DOI:** 10.3390/ani14243607

**Published:** 2024-12-14

**Authors:** Mark Arnold, Bryony Jones, Verity Horigan, Robin Simons, Brenda Rajanayagam

**Affiliations:** Department of Epidemiological Sciences, Animal and Plant Health Agency, Addlestone KT15 3NB, UK; bryony.jones@apha.gov.uk (B.J.); verity.horigan@apha.gov.uk (V.H.); robin.simons@apha.gov.uk (R.S.); brenda.rajanayagam@apha.gov.uk (B.R.)

**Keywords:** atypical scrapie, transmissible spongiform encephalopathy, Bayesian methods, transmission modelling, surveillance

## Abstract

Atypical scrapie (AS) is a transmissible spongiform encephalopathy (TSE) that affects sheep and goats. Low within-flock incidence suggests that AS is not transmissible between animals, and testing of all animals that exit positive flocks for two years following detection (i.e., intensified monitoring) used to be carried out in the EU to provide data to test this. This intensified monitoring stopped in 2021 but continues in Great Britain (GB). The aim of this study was to predict the number of AS cases missed if this monitoring were also stopped in GB, using a combination of statistical and transmission modelling. The number of AS cases estimated to be missed if the intensified monitoring was stopped was low relative to the number of AS cases detected in other active surveillance streams (e.g., fallen stock and abattoir surveys), at approximately 1 case every 3 years (0.34 per year, 95% CI: 0.18–0.54) compared to 10 per year (95% CI: 4–17) in the active surveillance stream. This suggests that stopping the intensive monitoring of AS would have relatively little impact on AS surveillance and on the power of the available AS data to infer whether AS is contagious.

## Simple Summary

Atypical scrapie (AS) is a disease of sheep and goats. Currently, when AS is detected on a farm in Great Britain (GB), all animals that exit the flock through natural death or slaughter are tested for AS for a period of 2 years following detection of the initial case, as used to be performed within the European Union (EU), in order to provide support as to whether AS can transmit directly between sheep. The aim of this study was to determine how many AS animals would be missed if this testing was also stopped in positive flocks in GB and hence determine the value of continuing with this surveillance programme. Our model estimated approximately one undetected AS case every three years if additional testing was stopped in detected flocks. In comparison, around 10 AS cases are detected per year through other active surveillance streams. Our model estimates suggest that stopping the intensive monitoring of AS would have relatively little impact on AS surveillance and that continuing it would provide limited evidence as to whether AS is contagious.

## 1. Introduction

Classical scrapie (CS) of sheep and goats is a contagious transmissible spongiform encephalopathy (TSE) where the normal prion protein (PrP^C^) misfolds into a pathogenic form (PrP^Sc^) in the central nervous system and also, in a proportion of cases, in the lymphoreticular tissues [1,2.,^3^]. CS was first described in sheep in Great Britain over 300 years ago [2,4] and is known to spread within sheep flocks, either by direct contact with infected animals or by contamination of the environment [5]. CS is not thought to pose any risk to humans. However, in the past, there were concerns that CS could mask a potential outbreak of bovine spongiform encephalopathy (BSE) in sheep [6,7] due to its similar clinical presentation to BSE in cattle, which does pose a risk to humans. This led to several legislative controls for CS across the European Union (EU). It was designated a notifiable disease in 1993, leading to the culling of all clinical suspects. Active surveillance in the form of annual testing of a sample of healthy sheep slaughtered for human consumption and fallen stock was introduced across the EU in 2002. In addition to this, targeted culling of high-incidence flocks and breeding programmes in some EU member states to increase genetic resistance to CS have also been undertaken.

Atypical scrapie (AS), first detected in 1998 [8], differs from CS in clinical signs, the distribution of PrP^Sc^ and the prion protein genotypes affected [9,10]. In contrast to CS, where within-flock clustering is evident [11], AS cases occur sporadically; cases usually occur as single events, and it is rare for a second case to occur in a given holding [12,13]. This apparent low within-flock incidence has led to uncertainty over whether AS is contagious. To address the question of whether AS is contagious and provide data to enable a greater understanding of flock-level risk factors and of the epidemiology of AS, in 2013, additional monitoring was introduced across the EU. This monitoring was applied to all flocks that experienced an AS case and required that all deceased (e.g., slaughtered, culled or died) ovine and caprine animals over the age of 18 months in the holding be tested for the presence of the TSE agent (intensified monitoring). Of the 742 flocks for which intensified monitoring was carried out across the EU, only an additional 35 AS cases were detected in 28 flocks (i.e., the majority of flocks had no additional AS cases detected). Also, the only flocks in which more than 1 additional case was detected were the two largest flocks: 2 additional cases for the largest flock (flock size approximately 15,000) and 7 additional cases for the second-largest flock (flock size approximately 13,000). A simulation model of within-flock AS transmission fitted to this EU-wide intensified monitoring data showed a better fit when assuming that AS was modelled as non-contagious compared to one assuming that it was contagious [1]. Furthermore, a statistical analysis showed that the occurrence of further cases (beyond the index case) in AS-positive flocks was not significantly higher than that of index cases across all EU flocks. It was therefore concluded that AS is more likely to be non-contagious [1]. As such, AS is recognized as a nonreportable disease separate to CS by the World Organization for Animal Health [9].

The intensified monitoring in AS-positive flocks was stopped across the EU in 2021 but is still mandatory in GB since GB (as part of the United Kingdom) left the EU in 2020. This monitoring programme can potentially provide further data to help elucidate flock-level risk factors for AS in sheep, help determine the likelihood that AS is contagious and provide material for experimental studies. On the other hand, the programme results in costs to farmers and governments in terms of the collection and disposal of animals, TSE testing and monitoring and reporting of results. This leads to the question of whether it is worthwhile to continue the intensified monitoring of AS-positive flocks in GB. Therefore, it is important to be able to estimate the number of additional cases that the intensified monitoring programme would detect so that an informed decision on whether it is worthwhile to continue with the monitoring programme can be made. The aim of this study was to provide statistical support to aid this decision by estimating the number of AS and CS cases in GB that would be missed if the intensified monitoring in AS-positive flocks was stopped, and by comparing these results with the number detected through other surveillance streams. A robust epidemiological interpretation of AS and CS data is made more difficult by the long incubation period of both CS and AS, with CS typically ranging from 2–7 years [12,14], and AS even longer [3,8,10]. Furthermore, the post mortem tests used are not able to detect scrapie until late in the incubation period [12]. To overcome these difficulties, statistical modelling that took into account the long incubation period and test sensitivity was used to infer the trend of AS in GB and the expected number of positive flocks per annum, and these estimates were combined with transmission modelling to predict the number of AS-positive sheep in flocks that qualify for intensified monitoring.

## 2. Materials and Methods

### 2.1. Data

#### 2.1.1. AS Data from Annual Sheep Surveys

The numbers of confirmed clinical suspects (i.e., passive surveillance) along with annual survey data were used in a back-calculation model of scrapie occurrence in GB to infer the true prevalence of AS. This annual survey data consisted of the number of sheep tested and the number of AS positives obtained from annual fallen stock and abattoir surveys, undertaken as part of TSE active surveillance requirements between 2005 and 2023 (Table 1). Positive AS cases were grouped by genotype, and these groupings followed the National Scrapie Plan (NSP) Types I–V, based on the level of resistance/susceptibility to CS [10,15]. These genotype groups were based on the 15 allelic variations at codons 136, 154 and 171 of the ovine PrP gene, with Type I being the most resistant to CS and Type V the least (Table 2). Genotype data were only available from a sample of the animals tested in the surveys. For the purposes of modelling, the observed genotype data were scaled up to generate total numbers sampled by genotype each year.

#### 2.1.2. Intensified Monitoring Data

Data were included from all flocks with AS cases for the entire period of intensified monitoring (i.e., 2011–October 2023). This consisted of 6605 animals tested from 143 farms, the majority of which were fallen stock (6178). During this period, five of the farms included in the intensified monitoring had a second AS case. The other surveillance streams in place in that period identified 178 AS cases (175 through active surveillance, 2 from passive surveillance and 1 detected from intensified monitoring triggered by a CS case in a flock); hence, intensified monitoring of AS has only identified a low proportion of the observed AS cases during its period of operation (Table 1).

### 2.2. Estimation of AS Infection Prevalence in GB: The Back-Calculation Model

The number of AS cases and, hence, the number of flocks for which intensified monitoring would be undertaken each year, was predicted using a statistical model. The model is based on one developed as part of a previous study, described in full in [12], but re-implemented in a Bayesian framework (Appendix A). The model inputs were the number of observed AS cases in 2005–October 2023 (Table 1), along with the size of the sheep population and age distribution and assumptions regarding the sensitivity of the diagnostic test for AS and the length of the incubation period (Appendix A). The Bayesian framework produced the probability distribution of the expected number of infected flocks (i.e., the model back-calculated the number of infected flocks that would result in the number of observed positive flocks). This estimate of the number of infected flocks was then used to predict the expected number of AS-positive flocks per year. The other parameters estimated by the model were as follows: the annual prevalence of AS, the relative risk of infection by genotype group (NSP groups I, II, IV and V relative to group III, as it was expected that the greatest risk of AS would occur in the AHQ and ARQ alleles [16]), the relative probability of being detected in fallen stock compared to healthy slaughter, and the degree of under-reporting of clinical suspects over time.

The annual prevalence for AS was estimated each year for the period 2005–2023, with the prevalence fitted to annual data on AS cases using an exponentially declining trend to take account of any possible reduction in the true prevalence of AS:

$$\pi_{t}=aexp(-bt)$$

where $\pi_{t}$ gives the prevalence in year *t* (*t* = 0 in 2005, *t* = 1 in 2006,…), *a* gives the initial infection prevalence in 2005 and *b* gives the exponentially declining annual trend. The estimate of $\pi_{t}$ was projected forwards one year (from 2023) to predict the number of sheep positive for AS in the fallen stock and abattoir survey streams for 2024, assuming the same number tested in those streams as in 2023. As the tests used for AS (and CS) are only expected to have high sensitivity towards the end of the incubation period [12], we also estimated the number of false-negative results (i.e., the number of undetected infected sheep).

For the parameters taking values between 0 and 1 (i.e., the four unknown genotype risks and the relative probability of being detected in fallen stock compared to healthy slaughter), minimally informative beta-distributed priors with both parameters equal to 1 were used (uniform between 0 and 1). For the other parameters (i.e., annual prevalence and under-reporting rate), normal priors were used with a mean of 0 and a precision of 0.001. All calculations were performed in WinBUGS 3.1 [17], using a burn-in of 5000 iterations followed by 5000 iterations of the model. Inspection of the history of each parameter and the Gelman–Rubin statistic [18] were used to check convergence.

### 2.3. Estimation of Expected AS Cases from Intensified Monitoring

The number of AS cases per year detected by intensified monitoring in a single flock was simulated using a within-flock transmission model, following the key assumptions for a similar model developed in a previous study [1]. The key model inputs and their sources were as follows:

- Flock size: generated by a bootstrap sample of the observed flock sizes of flocks that have been included in the intensified monitoring of AS in GB between 2011 and 2023.
- Number of sheep sent for human consumption and fallen stock.
- Observed number in each stream from the same flock that the flock size was obtained from.
- Within-flock transmission rate: sampled from the posterior distribution of a previously estimated between-sheep transmission rate [1].
- Sensitivity of the diagnostic test for AS relative to the proportion of the incubation period completed at the time of testing. This was assumed equal to that estimated for CS [12].
- Number of years before the first detection of AS in a flock that triggered the intensified monitoring. This estimate was based on the age of sheep in which AS was detected in GB. As was carried out in a previous study [1], a Weibull distribution was fitted to the age of all AS cases, and the resulting distribution was used to randomly generate the number of years prior to detection in the simulated flock.

Given these parameters, the model simulated the spread of AS within a flock and is described briefly below. Starting with one initial infection each year, the number of new infections was simulated based on the transmission rate and the number of sheep aged 0–1 in the flock (it was assumed that sheep became infected while young, in line with [1], and to match with what is known for CS [19,20]). Each new infection was assigned an incubation period randomly drawn from the lognormal distribution (Table A1) and an age at which it would leave the flock based on the overall age distribution (Table A1). At each yearly time step, the ages of the infected animals were increased and the transmission step repeated until the number of years passed was equal to the randomly drawn number of years prior to detection. At this point, one infected sheep was removed to represent the initial detection that triggered the intensified monitoring, and two further years of transmission were simulated. For each year, infected animals would exit the flock based on their simulated incubation period or age at exit, whichever was earliest (i.e., determining whether the animal reached clinical onset prior to exiting the flock), and would test positive with a probability depending on how long before clinical onset (Table A1). To generate the mean number of AS cases per year from the entire intensified monitoring programme, the number of positive flocks predicted for 2024 was taken from the back-calculation model. The number of AS cases was then calculated for 100 sets of 1000 runs, from which the mean and 2.5 and 97.5 percentiles were calculated.

Along with the number of AS cases from the intensified monitoring, it was also possible that there would be CS cases detected during the intensified monitoring of AS; hence, the expected number of CS cases was also estimated. This was carried out using a Bayesian version of a previously developed back-calculation model for CS [12], which uses the same input data as the AS model except it takes in numbers of CS cases in place of AS ones. It had the same priors as the AS model except the NSP group I genotype was set to equal 1 instead of group III. The mean number of sheep tested in the AS-positive flock intensified monitoring each year (by surveillance stream) was combined with estimates of the rate of CS cases by surveillance stream to derive an estimate for the expected number of CS cases in 2024.

## 3. Results

### 3.1. Estimation of AS Annual Prevalence in GB

**Figure 1:**
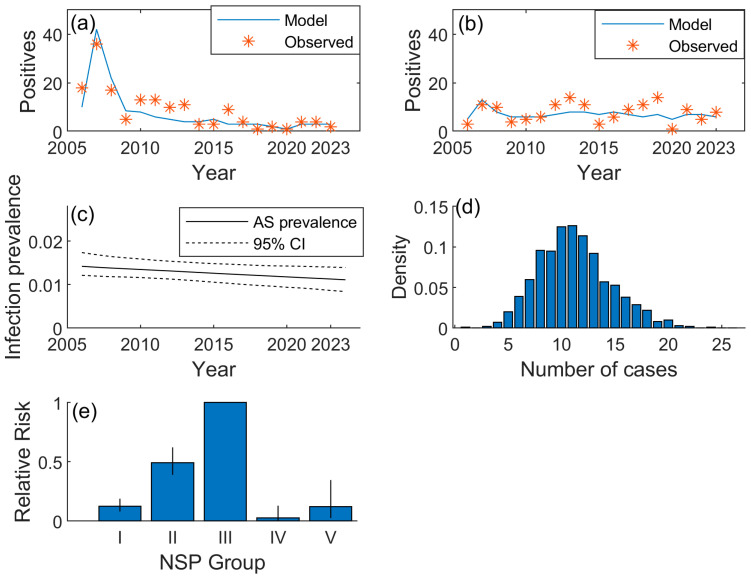
Outputs from a back-calculation model of atypical scrapie (AS) in Great Britain: (**a**) and (**b**) comparison between the model and observed data for the number of AS cases in the (**a**) annual abattoir survey and (**b**) annual fallen stock survey; (**c**) estimated true AS prevalence plus 95% CI; (**d**) distribution of the total number of AS cases from the abattoir and fallen stock surveys predicted for 2024; (**e**) estimated risk of infection relative to group III (plus error bars indicating 95% credible intervals) of AS by National Scrapie Plan genotype group.

There was a reasonable fit of the back-calculation model to the observed data (Figure 1a,b), where the model matched the decline in the number of AS sheep detected by the abattoir survey. The model matched the overall mean number detected in the fallen stock survey over time, although there was considerable variability in the number detected between years. The overall trend of AS cases has declined each year by 1.3% (95% CI: 0.11–3.1%) (Figure 1c) between 2005 and 2023, indicating that the numbers of AS cases detected in the intensified monitoring in the next few years would be expected to occur at a similar rate to those in recent years, since the rate of decline is low. Projecting the observed trend of AS cases forward by one year resulted in a predicted number of AS cases in 2024 equal to 10 (95% CI: 4–17) (Figure 1d), with the majority of the AS cases predicted to occur in the fallen stock survey (7 fallen stock positives, 95% CI: 2–13), with a median estimate of 3 abattoir survey cases (95% CI: 0–8). No passive surveillance cases were expected in 2024.

The number of false-negative AS sheep (i.e., those sheep that were tested too early in their incubation period to be detected by the test) was estimated to be greater in the fallen stock survey than the abattoir survey: 11 (95% CI: 5–19) and 7 (95% CI: 2–14) false negatives in the fallen stock and abattoir surveys, respectively. The estimated relative risk of infection by NSP genotype group (relative to Type III) showed that the greatest risk, after Type III, was for Type II, with relative risk equal to 0.50 (95% CI: 0.40–0.63). Types I and V had similar levels of relative risk to each other: Type I was equal to 0.12 (95% CI: 0.08–0.19) and Type V was equal to 0.13 (95% CI: 0.03–0.35). Type IV had the lowest relative risk, equal to 0.04 (95% CI: 0.001–0.13) (Figure 1e).

### 3.2. Estimation of Expected AS Cases from Intensified Monitoring

The simulations indicated a mean expected occurrence rate of 0.34 AS cases detected per year from the intensified monitoring (2.5 and 97.5 percentile range: 0.18–0.54).

The mean number of CS cases detected from the intensified monitoring was estimated to be 0.03 in 2024, suggesting that if CS prevalence remained at 2024 levels, there would be on average one detection of CS resulting from intensified monitoring every 33 years.

## 4. Discussion

Our simulation model predicted an expected rate of less than 1 AS case detected every 3 years in monitored flocks from continuing with the intensified monitoring. Given that the sampling of 793 flocks during the EU-wide intensified monitoring did not provide sufficient data to demonstrate conclusively whether AS was contagious, it is unlikely that continuing the intensified monitoring in GB only will collect enough data to reach a significant conclusion in the foreseeable future. In other words, with one additional case every 3 years from intensified monitoring, it would take many years to add sufficient power to the intensified monitoring data to demonstrate whether AS was contagious. One approach to enhancing the EU-wide analysis that might be worth exploring is the inclusion of flocks without AS into the analysis to increase the power; it should also be possible to perform this with GB data. The same approach adopted in a previous study [21] of using simulation models combined with approximate Bayesian computation could be applied, but the model for the non-contagious case would be enhanced by including data from flocks without AS, using annual census data.

A major source of uncertainty in the model estimates is that of the sensitivity of the diagnostic test for AS. The rapid test used was developed primarily for CS, and the assumed sensitivity of the test in the back-calculation model was based on data for CS [12]. For the detection of CS, the brainstem is the recommended area to be sampled. However, it has been found that the brainstem is a very poor area with regard to diagnostic sensitivity for AS [22,23,24], and, therefore, it has been recommended that sheep brain sampling should include the cerebellum in addition to the brainstem whenever possible. It is unclear from the case data the extent to which this has been conducted, hence the uncertainty in the test sensitivity for AS. An overestimation of the sensitivity of the diagnostic test for AS would result in a lower estimate of the true prevalence of AS (Figure 1c) and would also reduce the estimate of the number of false-negative AS-infected sheep. However, it would not impact the predicted number of AS cases detected from the active surveillance. This is because the model will adjust the estimated prevalence to compensate for any changes to the assumed sensitivity so that the expected number of cases in the model will match those observed. In other words, if the test sensitivity were halved, the prevalence estimate would double, and the resulting prediction of AS cases would remain unchanged. A similar effect would also be seen for the prediction of the number of AS cases detected through the intensified monitoring; a lower assumed sensitivity would result in a higher estimate of the transmission rate during model fitting to match the observed data, and the overall prediction of cases would be unaffected.

In predicting the number of CS cases that would be missed by the ending of the intensified surveillance, it was assumed that there was no correlation between the occurrence of both CS and AS on a farm. If there was a correlation between the occurrence of these scrapie types on a farm, then our model estimate would underestimate the number of CS cases in AS-positive flocks. However, a previous study assessing the co-occurrence of both of these scrapie types in GB flocks did not support an epidemiological link between them [3]; hence, even if there should be a correlation between them, it would be expected to be low and have only a minor impact on the estimated rate of CS occurrence in AS-positive flocks. The GB intensified scrapie monitoring data support the findings of this earlier study [3], as only one AS case was detected as part of the monitoring of CS-positive flocks.

The AS back-calculation model was fitted to the data using the genotype groupings defined by the NSP, which are based on observed cases by genotype for CS. The drawback of using the NSP genotype groupings is that it potentially includes relatively high-risk and low-risk genotypes in the same group, making it difficult to infer the risk by individual genotype. It has been shown that the alleles most associated with AS infection are AHQ and ARQ, [16,22,25,26] which are Group III according to the NSP genotype groupings, with the AS risk of the latter genotype additionally being increased by a polymorphism at codon 141 (F, phenylalanine) [9,25] to give the AFRQ allele. It would be possible to re-run the back-calculation model to estimate AS risks at the individual genotype level, although it is unclear whether the model would be able to produce results with sufficient certainty for the whole range of AS susceptible genotypes, as there would likely be too few samples for some of the genotypes. Such an exercise could then, with the addition of already published information on AS susceptibility by genotype, inform a more AS-specific set of genotype-based risk groups.

The simulation of AS within a holding was unable to take into account the role of genotype in between-sheep transmission, as there were no data on the genotype distribution within the flocks. The genotypes of the sheep within a flock will have an impact on the likelihood of AS cases, and the lack of genotype data could impact the reliability of the predictions of future AS cases if the genotype distribution of flocks that enter the GB intensified monitoring differ significantly from those sampled in the EU-wide monitoring (i.e., those from which the transmission rate was estimated). However, the predicted rate of future cases of AS from continuing intensified monitoring in GB (one case per 3 years) was similar to the rate observed in GB monitoring between 2011 and 2023 (five cases in 13 years) using the transmission rate estimated from the EU-wide intensified monitoring.

The back-calculation model inferred a statistically significant decline in AS prevalence in GB between 2011 and 2023. Previous studies looking at the trend of AS prevalence in GB have not detected any change over time [3,27], although these were conducted using fewer years of data. More recent data indicate a mean 4% annual decline in the number of AS cases detected in EU member states between 2012 and 2023 [28]. It remains unknown why AS prevalence has decreased through time, and one would expect the annual prevalence of a sporadic non-contagious disease to remain constant. One possibility is that the genotype profiles of GB and EU sheep are changing over time in response to breeding for CS resistance. However, breeding programs across the EU have favoured group II genotypes, some of which appear to be more susceptible to AS. It is also possible that there are biases in those animals that are tested in the annual surveys to determine AS prevalence (e.g., only certain flocks tested or the age distribution of the animals tested). For example, a previous study found biases in those sheep sampled for active surveillance in GB, with larger flocks more likely to be over-represented [3]. It is also unknown whether there is any bias in the genotypes of the animals sampled compared to the general population. It is problematic for the back-calculation model to account for these biases. However, the temporal trend estimated by the model, although statistically significant, is very low and has only a small impact on the predicted number of cases arising from continuing with the intensified monitoring in GB.

For the predictions of future AS cases detected by intensified monitoring in GB, it was assumed that AS is contagious. Assuming AS is non-contagious would have little impact on the predicted number of AS cases detected from GB-wide intensified monitoring. This is because a previous study found it could match the observed rate of occurrence in the EU-wide intensified monitoring similarly with models assuming AS is contagious or non-contagious; being contagious or not only influences how AS is distributed between flocks. However, if AS is contagious, then it does have important implications for the control and surveillance of AS. Firstly, if contagious, then AS would increase in prevalence over time in susceptible flocks due to within-flock transmission, meaning that early detection in those flocks is important. Secondly, the effective identification of infected flocks and controls in those flocks could potentially lead to the elimination of AS (subject to effective surveillance), whereas if non-contagious, then AS will be problematic to eliminate from the national population without a breeding programme to promote AS-resistant genotypes.

## 5. Conclusions

Our models estimated only one undetected case of AS every three years if the intensified monitoring program in GB ended. This value is statistically lower than the number of AS cases detected by other active surveillance streams (10 per year) but higher than the number expected from passive surveillance (none expected). In the context of these figures, it appears that stopping the intensified monitoring of AS would have relatively little impact on AS surveillance and on the power of the available AS data to infer whether AS is contagious. There would be value in further studies looking at the sensitivity of rapid tests for the detection of AS, on the relative risk of infection/transmission of AS by genotype and on reanalysis of the GB data for AS intensified monitoring, including negative flocks.

## Figures and Tables

**Table 1:** Summary of the annual number of confirmed cases of scrapie in sheep for classical and atypical scrapie in Great Britain by surveillance stream.

Year	Classical Scrapie			Atypical Scrapie		
	Passive	Active	Intensified Monitoring ^1^	Passive	Active	Intensified Monitoring ^1^
**2005**	178	43	3	0	22	-
**2006**	97	44	3	0	47	-
**2007**	10	23	2	0	31	-
**2008**	1	7	0	0	10	-
**2009**	3	5	0	0	25	-
**2010**	0	1	0	0	19	-
**2011**	44 *	5	0	0	22	0
**2012**	0	2	4 **	0	28	0
**2013**	0	3	2 ***	1	16	0
**2014**	0	0	0	0	10	0
**2015**	0	2	0	0	15	2
**2016**	0	0	0	0	13	1
**2017**	0	0	0	0	12	0
**2018**	0	0	0	0	16	1
**2019**	0	1	8 ***	0	6	0
**2020**	0	0	0	0	13	1
**2021**	0	0	0	0	11	0
**2022**	0	0	0	1	10	0
**2023**	0	0	0	0	3	0
**Total positive**	111	136	22	2	329	5
**Total tested**	N/A	416, 368	2959	N/A	416, 368	6605
**Percent positive**	N/A	0.033%	0.74%	N/A	0.079%	0.076%

Data valid to 31 October 2023. ^1^ Also known as Compulsory Scrapie Flock Scheme. Data separated into CS and AS triggered surveillance from 2011, prior to the start of the EU AS-specific intensified monitoring. * A total of 42 out of the 44 classical positive passive cases were from a single flock. ** Two cases from the same holding. *** All cases from the same holding.

**Table 2:** Risk groups by genotypes for classical scrapie in sheep (based on [15], used also in the back-calculation model of atypical scrapie).

Genotype	National Scrapie Plan Risk Group	Associated Classical Scrapie Risk
ARR/ARR	I	Very Low
ARR/AHQ	II	Low
ARR/ARH		
ARR/ARQ		
AHQ/AHQ	III	Moderate
AHQ/ARH		
AHQ/ARQ		
ARH/ARH		
ARH/ARQ		
ARQ/ARQ		
ARR/VRQ	IV	Moderate (but greater than Type III)
AHQ/VRQ	V	High, especially ARQ/VRQ and
ARH/VRQ		VRQ/VRQ
ARQ/VRQ		
VRQ/VRQ		

## Data Availability

The original contributions presented in this study are included in the article/Supplementary Materials. Further inquiries can be directed to the corresponding authors.

## Supplementary Materials

The following supporting information can be downloaded at: https://www.mdpi.com/article/10.3390/ani14243607/s1.

## Appendix A. Description of Animal-Level Back-Calculation Model

**Table A1:** List of model parameters used in a Bayesian model to infer the true prevalence of atypical scrapie infection in Great Britain from passive and active surveillance data.

Parameter	Description	Value	Source
f	Incubation period distribution	Lognormal (4.47, 0.3) (years)	[1]
S(a)	Probability of sheep survival until age *a*	Sheep flock population by age for GB Results in a replacement rate of 20.2%	[29]
ω(a)	Proportion of sheep of age *a* that are found dead on farm	0.726 (aged 1–2 years), 0.335 (2–3 years), 0.216 (3–4 years), 0.198 (4–5 years), 0.147 (6–8 years), 0.11 (aged 9 years) and 0.12 (10 years and over)	[14]
ψ(t)	Sensitivity of diagnostic test *t* months prior to clinical onset	Based on estimated incubation period and experimental data for CS; this results in 98% sensitivity for up to 12 months before onset, 75% for 13–24 months, 38% for 25–36 months and 13% for 37–48 months	[12]
ρ	Reporting rate of clinical sheep	Estimated by model	N/A
K	Probability that infected sheep end up in healthy slaughter stream	Estimated by model	N/A
*r_j_*	Relative risk of scrapie infection by genotype group *j*	Estimated by model by NSP genotype group; Type III (highest risk) set to 1	N/A
*θ_t_*	Baseline risk of infection by year *t*	Estimated by model	N/A

There were three exit streams for scrapie-infected sheep:

- Detected in the fallen stock survey, with probability denoted as *P_FS_*;
- Reported as a clinical suspect (passive surveillance), with probability denoted as *P_C_*;
- Detected in the abattoir survey, with probability denoted as *P_AS_*.

It was assumed that the number of sheep positive in year *j* in each of the three streams followed a binomial distribution with parameters *p* given by *P_FS_*, *P_C_* and *P_AS_* and n given by the number of fallen stock tested, the size of the sheep population and the number of abattoir survey sheep tested, respectively, for fallen stock, clinical suspects and the abattoir survey. The calculation of *P_FS_*, *P_C_* and *P_AS_* is described below, using the notation in Table A1.

### Appendix A.1. Probability of Detection in Fallen Stock

There were two possible ways that an animal could end up as a fallen stock positive at age *a*: (i) it reached clinical onset without being identified by the farmer and died of scrapie, or (ii) it died on the farm (not due to scrapie) and happened to have scrapie. Probability (i) is the product of the probability that the sheep reached clinical onset and was not reported (denoted by 1 − ρ) (e.g., due to lack of farmer awareness of how to spot the clinical signs of scrapie), the risk of infection of genotype *j*, (*r_j_*), the probability of surviving to age *a (S (a))*, and the probability that it reached the end of its incubation period at age *a (f (a))*. Probability (ii) is the product of the risk of infection by genotype (*r_j_*), the proportion of animals of age *a* that are found dead on the farm (=ω(a)(*S*(*a*) − *S*(*a* + 1)), and the likelihood that an infected animal of age *a* will be detected by the diagnostic test (which depends on the sensitivity of the test (ψ). We also allow the potential for differential slaughter of sub-clinically affected sheep, where sub-clinical sheep may be at higher risk of slaughter due to an effect of scrapie on their production traits, so that sheep end up in the fallen stock/clinical stream with probability 1 − *K*, and in the healthy slaughter stream with probability *K*. Therefore, the probability of being detected as a fallen stock positive at age *a* is given by

$$F_{FS}=\theta \sum_{j}r_{j}(1-K)\left\{S(a)f_{j}(a)+\omega (a)(S(a)-S(a+1))\int_{a}^{\infty }f_{j}(x)\psi (x-a)dx\right\}$$

where *θ* is the baseline risk of infection and ψ(t) is the probability that the diagnostic test will detect an infected animal *t* months before clinical onset.

### Appendix A.2. Probability of Detection as a Clinical Suspect

The probability that an animal would end up as a clinical suspect was given by

$$F_{C}=\theta \sum_{j}r_{j}(1-K)\rho S(a)f_{j}(a)$$

To account for possible changes in both the baseline prevalence and the rates of reporting of scrapie over time, possibly due to changes in farmer awareness as disease prevalence declines, both were allowed to vary each year, i.e., $\theta =a_{1}\exp\left(-b_{1}t\right)$ and $\rho =a_{2}\exp\left(-b_{2}t\right)$, where *a*_1_, *b*_1_, *a*_2_, and *b*_2_ are parameters to be estimated and *t* is the number of years since 2005 (the start of the model fitting).

### Appendix A.3. Probability of Detection Through the Abattoir Survey

The probability that an animal would be detected as an abattoir survey positive was given by the product of the risk of infection, the proportion of animals of age *a* that are sent for slaughter (i.e., do not die on the farm) (1 − ω(a))(*S*(*a*) − *S*(*a* + 1)) and the likelihood that an infected animal of age *a* will be detected by the diagnostic test. Therefore, the probability of being detected as an abattoir survey positive at age *a* is given by

$$P_{AS}=\theta \sum_{j}r_{j}K\left\{(1-\omega (a))(S(a)-S(a+1))\int_{a}^{\infty }f_{j}(x)\psi (x-a)dx\right\}$$
